# Successful hemostasis with red dichromatic imaging for bleeding rectal dieulafoy’s lesion

**DOI:** 10.1055/a-2253-8797

**Published:** 2024-02-15

**Authors:** Chih-Wen Huang, Hsu-Heng Yen, Yang-Yuan Chen

**Affiliations:** 1 36596 Division of Gastroenterology, Changhua Christian Hospital, Changhua, Taiwan; 2 34916 Department of Post-Baccalaureate Medicine,College of Medicine, National Chung Hsing University, Taichung, Taiwan

A 74-year-old man with a history of type 2 diabetes mellitus and hemodialysis suffered from bloody stool for 2 days and underwent a colonoscopy. Laboratory data showed a drop in hemoglobin to 7.2 g/dL. During the colonoscopy, fresh blood was observed in the rectum, where a Dieulafoy's lesion was actively bleeding. Under the white light image, the intestinal wall was coated with fresh blood, making it difficult to clearly identify the bleeding point ( Fig. 1 , Video 1 ). We switched to the red dichromatic imaging (RDI) mode for further examination, which allowed us to accurately locate the bleeding point and perform hemostasis ( Fig. 2 ). In the end, the hemostasis of Dieulafoyʼs lesion was successfully achieved ( Fig. 3 ).

**Fig. 1:**
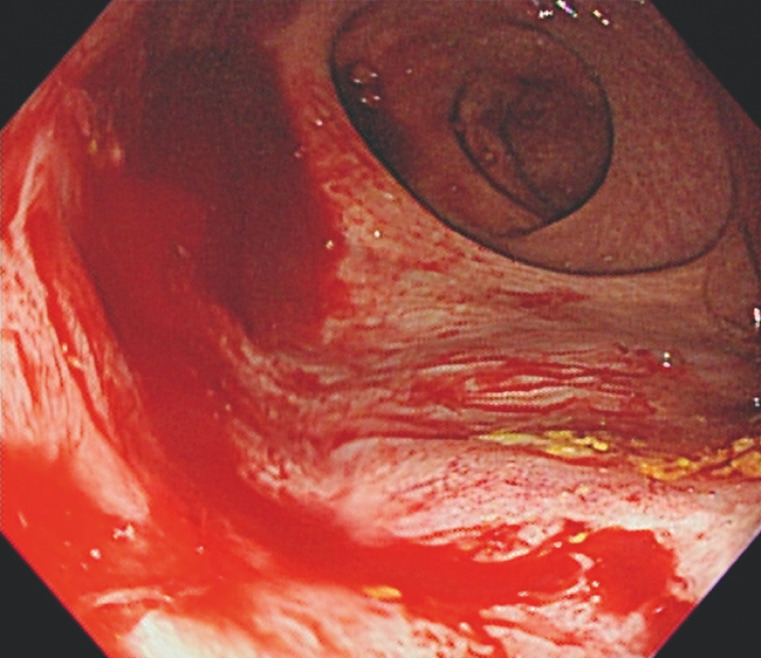
Active bleeding was found from the rectal Dieulafoy’s lesion.

There is active bleeding found in the rectum. Under red dichromatic imaging mode, hemostasis was facilitated by the easy separation of the active bleeder and the surrounding blood pool.Video 1

**Fig. 2:**
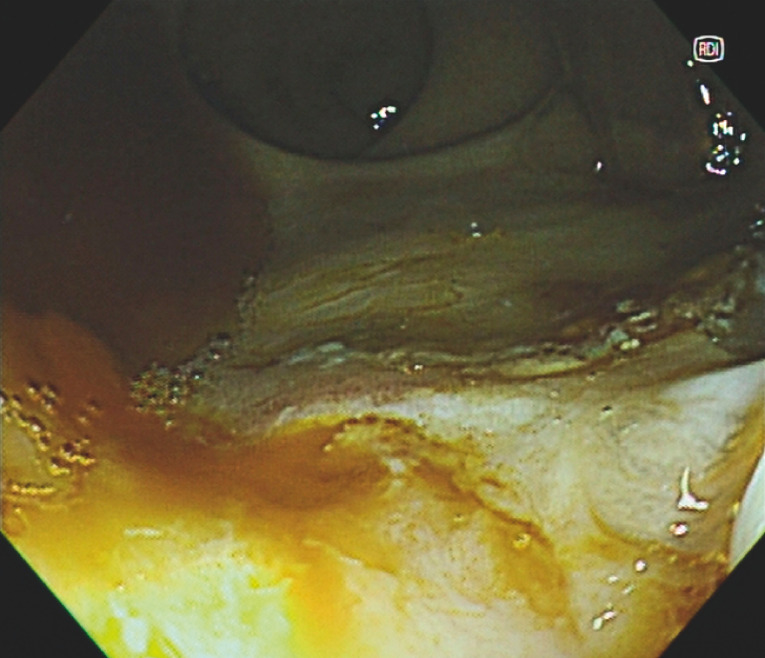
The bleeding point was accurately located after switching to red dichromatic imaging mode.

**Fig. 3:**
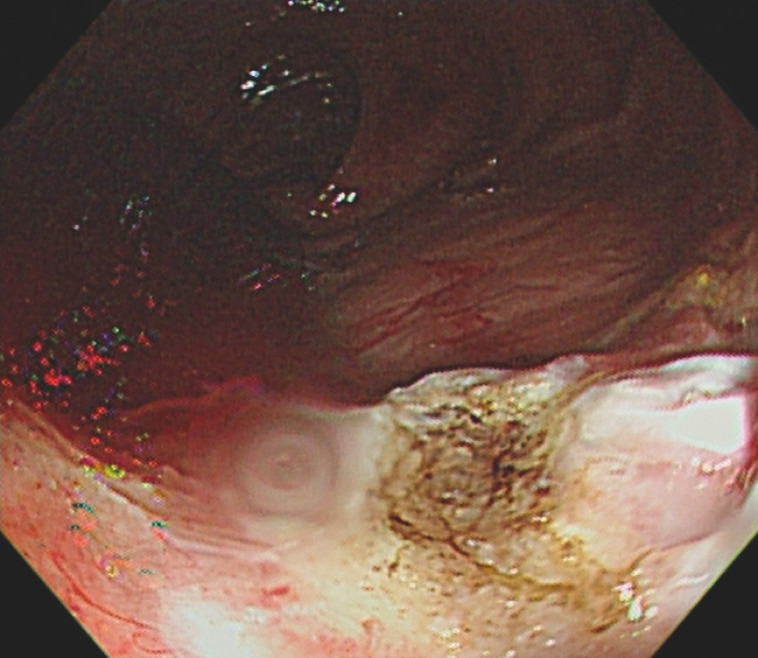
Endoscopic view of successful hemostasis.

RDI is part of a new-generation image-enhanced endoscopy system (EVIS X1; Olympus Marketing, Tokyo, Japan). It utilizes both red and amber wavelengths (600 nm and 630 nm) to enhance the color contrast between the bleeder and surrounding blood pool and facilitate the identification of bleeding sites 1 . Previous reports have demonstrated its usefulness to facilitate endoscopic submucosal dissection procedures. Furthermore, the actual bleeder of a peptic ulcer 2 or post-sphincterectomy 3 was identified rapidly and precisely in RDI mode.

Dieulafoy’s lesion is an uncommon cause of acute gastrointestinal bleeding 4 . In this case, RDI could effectively assist in hemostasis, especially for inexperienced endoscopists in the emergency setting.

Endoscopy_UCTN_Code_CCL_1AD_2AF

## Notes

Endoscopy E-Videos https://eref.thieme.de/e-videos
*E-Videos* is an open access online section of the journal *Endoscopy* , reporting on interesting cases and new techniques in gastroenterological endoscopy. All papers include a high-quality video and are published with a Creative Commons CC-BY license. Endoscopy E-Videos qualify for HINARI discounts and waivers and eligibility is automatically checked during the submission process. We grant 100% waivers to articles whose corresponding authors are based in Group A countries and 50% waivers to those who are based in Group B countries as classified by Research4Life (see: https://www.research4life.org/access/eligibility/ ). This section has its own submission website at https://mc.manuscriptcentral.com/e-videos .
